# Electric-field-controlled interface dipole modulation for Si-based memory devices

**DOI:** 10.1038/s41598-018-26692-y

**Published:** 2018-05-31

**Authors:** Noriyuki Miyata

**Affiliations:** National Institute of Advanced Industrial Science and Technology (AIST), Central 5, 1-1-1 Higashi, Tsukuba, Ibaraki, 305-8565 Japan

## Abstract

Various nonvolatile memory devices have been investigated to replace Si-based flash memories or emulate synaptic plasticity for next-generation neuromorphic computing. A crucial criterion to achieve low-cost high-density memory chips is material compatibility with conventional Si technologies. In this paper, we propose and demonstrate a new memory concept, interface dipole modulation (IDM) memory. IDM can be integrated as a Si field-effect transistor (FET) based memory device. The first demonstration of this concept employed a HfO_2_/Si MOS capacitor where the interface monolayer (ML) TiO_2_ functions as a dipole modulator. However, this configuration is unsuitable for Si-FET-based devices due to its large interface state density (*D*_*it*_). Consequently, we propose, a multi-stacked amorphous HfO_2_/1-ML TiO_2_/SiO_2_ IDM structure to realize a low *D*_*it*_ and a wide memory window. Herein we describe the quasi-static and pulse response characteristics of multi-stacked IDM MOS capacitors and demonstrate flash-type and analog memory operations of an IDM FET device.

## Introduction

Emerging memory devices with various mechanisms have been investigated in an effort to replace Si-based NAND flash memories, which are the most common type of digital storage device, *e*.*g*., resistive random access memories (ReRAMs), phase change memories (PCMs), ferroelectric tunnel junctions (FTJs), and ferroelectric field-effect transistors (FeFETs)^1–8^. The advantages of these new devices are a faster operation speed and a higher endurance than a conventional flash memory. Material compatibility with conventional Si device technologies provides a competitive advantage to realize mass production. In particular, Si metal-oxide semiconductor (MOS) FETs used for flash memories are promising building blocks as they provide a high-density three-dimensional memory-cell platform, which can reduce the development cost.

In a flash memory, the electric charge accumulated in the gate stack structure of the MOSFET is read out as the channel current. Similarly, FeFETs utilize the spontaneous polarization of a ferroelectric material integrated in the MOS structure. In particular, ferroelectric HfO_2_ is a promising material in terms of Si material compatibility because Hf-based gate oxides are employed in advanced Si complementary MOS devices^7,8^. Various elaborate methods have been proposed to fabricate orthorhombic HfO_2_ that acts as a ferroelectric, including doping with Zr, Si, Al, Y, Gd, La, or Sr, and controlling the thermal budget of pure HfO_2_ films^9–12^. Indeed, a flash-type memory operation of HfO_2_-based FeFET arrays fabricated with an advanced Si-CMOS platform has been demonstrated^8^. Therefore, it is worthwhile to explore HfO_2_-based memory materials that are compatible with Si CMOS technology.

Many recent studies have focused on the continuous conductance changes of memory devices because such a change should realize high-efficiency low-power neuromorphic computing^13–17^. The spike-timing-dependent plasticity (STDP) is the most common biological synaptic learning rule. In STDP, the synaptic weight varies with the time difference between presynaptic and postsynaptic neuron spikes. In electronic memory-based synapse devices, electrical pulses (*e*.*g*., pulse number, voltage amplitude, width, and polarity) control the conductance change and are utilized to emulate the STDP behavior. As examples, the electric-field-controlled conductive filament in a ReRAM device, the thermally controlled amorphous/crystalline phase change in a PCM device, and the field-controlled ferroelectric domain growth in the FTJ and FeFET devices have been utilized for the STDP operation^13–17^. From the viewpoint of Si-MOS compatibility, HfO_2_-FeFET devices are advantageous^17^, and the new memory device proposed in this paper has similar advantages.

Herein firstly, a new memory concept, interfacial dipole modulation (IDM) occurring at HfO_2_/Si and HfO_2_/SiO_2_ interfaces, is explained and demonstrated. Then the flash-type memory operation and pulse-induced gradual current change of Si-FET-based IDM device are reported.

**Figure 1:**
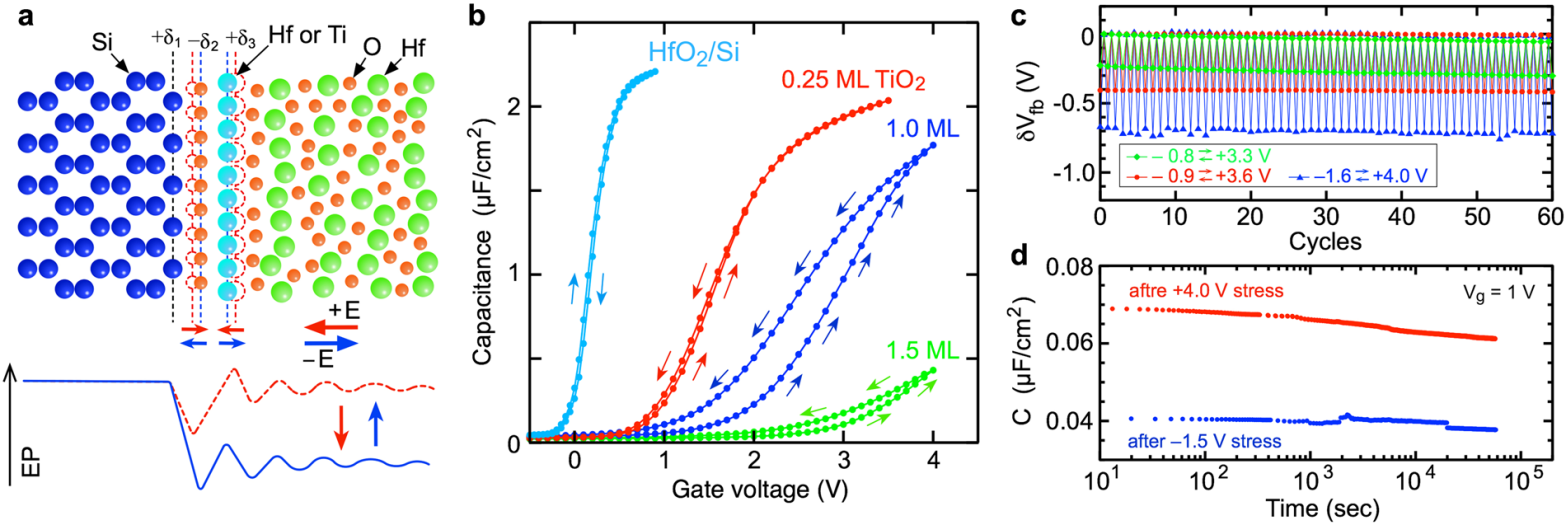
Interface dipole modulation (IDM) concept and experimental demonstration at the HfO_2_/Si interface. **(a**) Schematic illustration of dipole formation and IDM operation at the HfO_2_/Si interface. Large interface dipoles are observed from the HfO_2_/Si structures, which are reasonably explained by the bond polarity mechanism, from ref.^29^. The IDM operation utilizes this mechanism. Dipole strength changes due to the atomic displacement induced by the electric field. (**b**) Effect of the interface TiO_2_ layer on the high-frequency capacitance-voltage (*C-V*) curves of the 5.5-nm-thick HfO_2_/Si MOS capacitors fabricated on n-type Si substrates. Double sweep measurements were performed in the voltage ranges between −1.0 V and the positive voltages shown in this figure. Counterclockwise hysteresis suggests that the IDM behavior appears for the HfO_2_/1-monolayer (ML) TiO_2_/Si structure. (**c)** Cyclic operation of the HfO_2_/1-ML TiO_2_/Si IDM device under various voltage sweep conditions. (**d**) Retention characteristics of the HfO_2_/1-ML TiO_2_/Si IDM device. Large and small capacitance states measured at +1.0 V are plotted as a function of time after applying a stress voltage.

Controlling interfacial dipoles affects the interface band alignment and is indispensable in the development of semiconductor devices. Thus, dipole formation at a solid/solid interface is well researched. Numerous dipole formation mechanisms have been proposed for metal/semiconductor, semiconductor/semiconductor, and oxide/semiconductor interfaces. These mechanisms are roughly classified into two models: charge transfer due to the interface states and electric polarization of the interface chemical bonds^18–22^. Recently, the interface dipoles formed in HfO_2_-based stack structures are well studied because they are related to threshold voltage control of the HfO_2_/Si MOSFETs^23–27^. However, the dipole formation in the gate stack structures including the oxide/oxide interfaces is complicated compared to the above interfaces. As the simplest structure, we previously reported that a large dipole (>0.5 V) is formed at the HfO_2_/Si interface^28^, and proposed a bond polarity mechanism in which positive and negative alternating charged atoms produce a large potential difference between the HfO_2_ and Si sides, as shown in Fig. 1a ^29,30^. In this model, the polarizations of the interfacial Si-O and O-Hf bonds largely affect the total potential difference because these interface regions have small predicted local dielectric constants compared to Si and HfO_2_. This is easily predicted from microscopic dielectric responses based on the chemical gradient^31^. Thus, interface chemical bonding largely influences MOSFETs operations, depending on the strength of interface dipole^32^.

The IDM concept originates from the above dipole formation mechanism at the HfO_2_/Si interface. If the electric field induced by the gate voltage changes the position of the interface atoms, the interface dipole is supposed to be modulated (Fig. 1a). Since similar switchable interfacial metal-oxygen bonds have been predicted for metal/ferroelectric oxide interfaces using first principles calculations^33^, we expect that the IDM operation occurs at the HfO_2_/Si interface if an appropriate interface bonding is constructed. The IDM-integrated FET (IDM FET) is expected to behave like FeFET. However, the operation mechanism is completely different. Although switching of spontaneous polarization in a ferroelectric film is utilized in FeFETs, modulation of the dipoles induced in the atomic-scale interface region dominates IDM operation.

The double swept capacitance-voltage (*C-V*) trace of the conventional HfO_2_/Si MOS capacitor in Fig. 1b does not show any evidence of the IDM operation. A small clockwise hysteresis takes place, indicating charge trapping around the HfO_2_/Si interface^34^. Meanwhile, a counterclockwise hysteresis appears when a monolayer-thick TiO_2_ is inserted at the HfO_2_/Si interface. A particularly large hysteresis (>0.5 V) occurs at the 1-ML TiO_2_ interface. A counterclockwise hysteresis indicates charge movement inside the gate stack structure, that is, ferroelectric polarization inversion^6^ or an IDM operation. Since the HfO_2_ layer is amorphous (Supplementary Fig. S1a), the ferroelectric effect can be excluded.

The hysteresis width strongly depends on the insertion of monolayer TiO_2_. It may be reasonable to consider that some structural change of the interface TiO_2_ produces a large potential change between the Si and HfO_2_ sides. In this manuscript, we call the interface 1-ML TiO_2_ a dipole modulator. The fundamental memory function, cyclic modulation, and two-states retention are obtained (Fig. 1c and d). The modulation width strongly depends on the sweep voltage range, and reaches about 0.7 V. This value is comparable to the intrinsic interface dipole observed for the HfO_2_/Si interface^28–30^. The long retention can exclude the effect of electron and/or hole trapping around the interface because the typical interfacial-charge trapping shows a much shorter response^34,35^. On the other hand, the *C-V* curve for the 1-ML TiO_2_ sample is stretched towards a positive bias, indicating that the interface state density (*D*_*it*_) is larger than that of non-TiO_2_ interface. Actually, *D*_*it*_ of the HfO_2_/TiO_2_/Si IDM structure is estimated to be about 2 × 10^13^ cm^−2^ eV^−1^ around the mid-gap energy (Supplementary Fig. S2). This is easily predictable since an electrically switchable TiO_2_ layer likely includes large amounts of unstable bonds. Thus, the HfO_2_/Si IDM structure is not suitable for FET-based memory devices.

**Figure 2:**
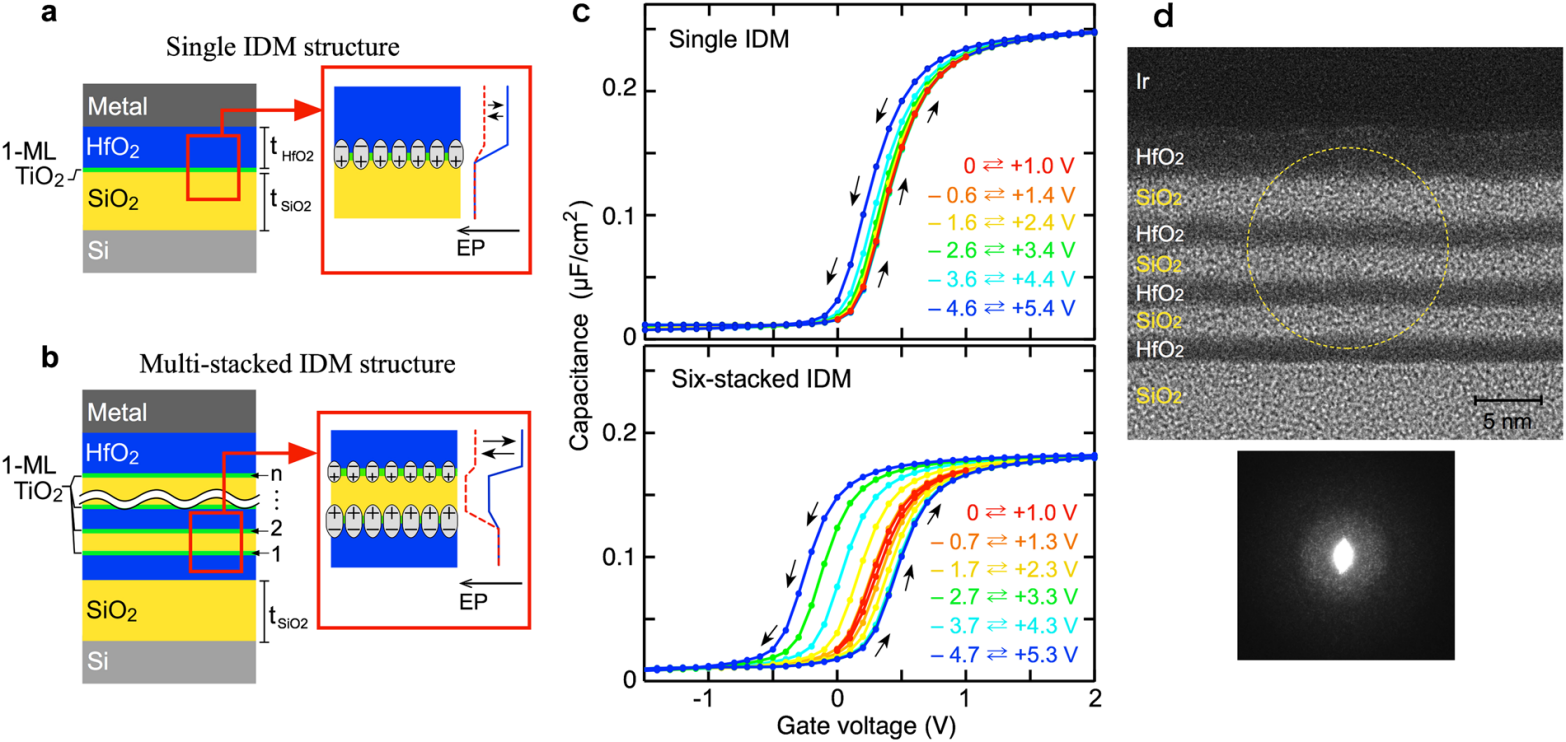
HfO_2_/SiO_2_-based IDM structures and *C-V* hysteresis curves. (**a** and **b)** Schematic illustration of single and multi-stacked HfO_2_/1-ML TiO_2_/SiO_2_ MOS structures. According to the IDM mechanism, the modulation by the TiO_2_ modulators at two facing interfaces are superimposed and enhanced. (**c**) *C-V* curves observed from single and six-stacked IDM MOS capacitors fabricated on n-type Si substrates. The former consists of 3-nm-thick top HfO_2_ and 10-nm-thick bottom SiO_2_ layer, while the latter consists of 3.5-nm-thick top HfO_2_, 1.8-nm-thick inner SiO_2_, 1.8-nm-thick inner HfO_2_, and 10-nm-thick bottom SiO_2_ layers. Small counterclockwise hysteresis appears in a single IDM structure and an obviously large hysteresis appears in the six-stacked IDM structure. (**d**) Transmission electron microscopy (TEM) image and electron diffraction (ED) pattern of the six-stacked IDM structure. These results indicate that all oxide layers are amorphous. Note that it is difficult to distinguish between SiO_2_ and TiO_2_ in the TEM image.

We then explored HfO_2_/SiO_2_-based IDM structures to solve the interface state problem since inserting a SiO_2_ layer should separate the charge traps created around the TiO_2_ modulator from the Si surface. Experimental and theoretical studies have been conducted on dipole formation at HfO_2_/SiO_2_ interfaces. The proposed mechanisms are somewhat complicated compared to those for metal/semiconductor, oxide/semiconductor interfaces, etc. However, most mechanisms for dipole formation at HfO_2_/SiO_2_ interfaces are based on charge transfer occurring at the HfO_2_/SiO_2_ interface such as an electronegativity effect around interfacial Hf-O-Si bonding and the movement of oxygen atoms^24–27^. Therefore, we expect an IDM operation in the HfO_2_/SiO_2_ stack structure when some of the charged atoms around the HfO_2_/SiO_2_ interface are moved by the gate bias. The HfO_2_/1-ML TiO_2_/SiO_2_ IDM structure (Fig. 2a) exhibits a hysteresis *C-V* curve without displaying stretched-out characteristics (Fig. 2c). Actually, *D*_*it*_ estimated for this MOS structure is comparable to that of conventional SiO_2_/Si interface (*D*_*it*_ < 1 × 10^11^ cm^−2^ eV^−1^ around the mid-gap energy, Supplementary Fig. S2c). The *C-V* curve shows a counterclockwise hysteresis as well as the features of the above HfO_2_/Si IDM structure. It is obvious that carrier injection from the Si substrate is not the origin of hysteresis. Meanwhile, carrier injection from the gate electrode, that is, carrier trapping by interface TiO_2_ potentially results in a counterclockwise hysteresis. However, this mechanism should also be excluded because the hysteresis characteristic is independent of the thickness of the top HfO_2_ layer, as explained below. On the other hand, the hysteresis window is obviously small (<0.2 V) and insufficient for memory applications. This probably originates with the difference in the intrinsic dipole strength; that is, the dipole of the HfO_2_/SiO_2_ interface is reported to be smaller than that of the HfO_2_/Si interface^26,28^. One reason for this smaller dipole may be due to disordered chemical bonding at amorphous HfO_2_/SiO_2_ interfaces.

Amorphous materials can be easily stacked (Fig. 2b). Thus, multi-TiO_2_ modulators can be integrated in the same MOS structure. Here, we consider two types of IDM behavior: the upper-HfO_2_/lower-SiO_2_ and the upper-SiO_2_/lower-HfO_2_ interfaces. Under an electric field induced by a positive gate bias, the former and the latter dipoles are predicted to increase and decrease, respectively. Under the opposite electric field, the opposite dipole modulations occur. This means that the TiO_2_ modulations occurring at two facing interfaces are superimposed and contribute to the enhanced memory window. In fact, a larger hysteresis is observed from the six-stacked HfO_2_/1-ML TiO_2_/SiO_2_ IDM structure (Fig. 2c). Thus, the multi-stacked IDM structure is preferable for memory application.

The transmission electron microscopy (TEM) image and electron diffraction pattern (Fig. 2d) exhibit that the HfO_2_ layers are amorphous. Therefore, the effect of ferroelectric HfO_2_ can also be eliminated, even for a multi-stack HfO_2_/SiO_2_ IDM structure. In general, the formation of a ferroelectric HfO_2_ film requires annealing at a temperature above 450 °C and the thinnest HfO_2_ film employed in their experiments is 5 nm^7–12,17,36,37^. The annealing temperature of the six-stacked IDM structure shown in Fig. 2d is 350 °C, and the thickness of internal HfO_2_ layer is 1.8 nm. It was reported that HfO_2_ crystallization hardly occurs when the film thickness becomes thin^38^. Thus, we can reasonably conclude that our IDM structure does not include crystalline HfO_2_. In addition, the memory window of the single HfO_2_/1-ML TiO_2_/SiO_2_ IDM structure is independent of the HfO_2_ film thickness, as mentioned below. This means that, rather than bulk HfO_2_ (*i*.*e*., a ferroelectric effect), the interface is a major component in the IDM operation. On the other hand, the HfO_2_/SiO_2_ interface shown in the TEM image has atomic-scale roughness, indicating that various bonding configurations probably exist at this interface. In the IDM operation, the charge displacement component perpendicular to the interface is considered to contribute to the potential change. The atomically rough interface is probably disadvantageous for the IDM operation. Consequently, if an atomically abrupt interface is formed, the memory window should be further enhanced.

**Figure 3:**
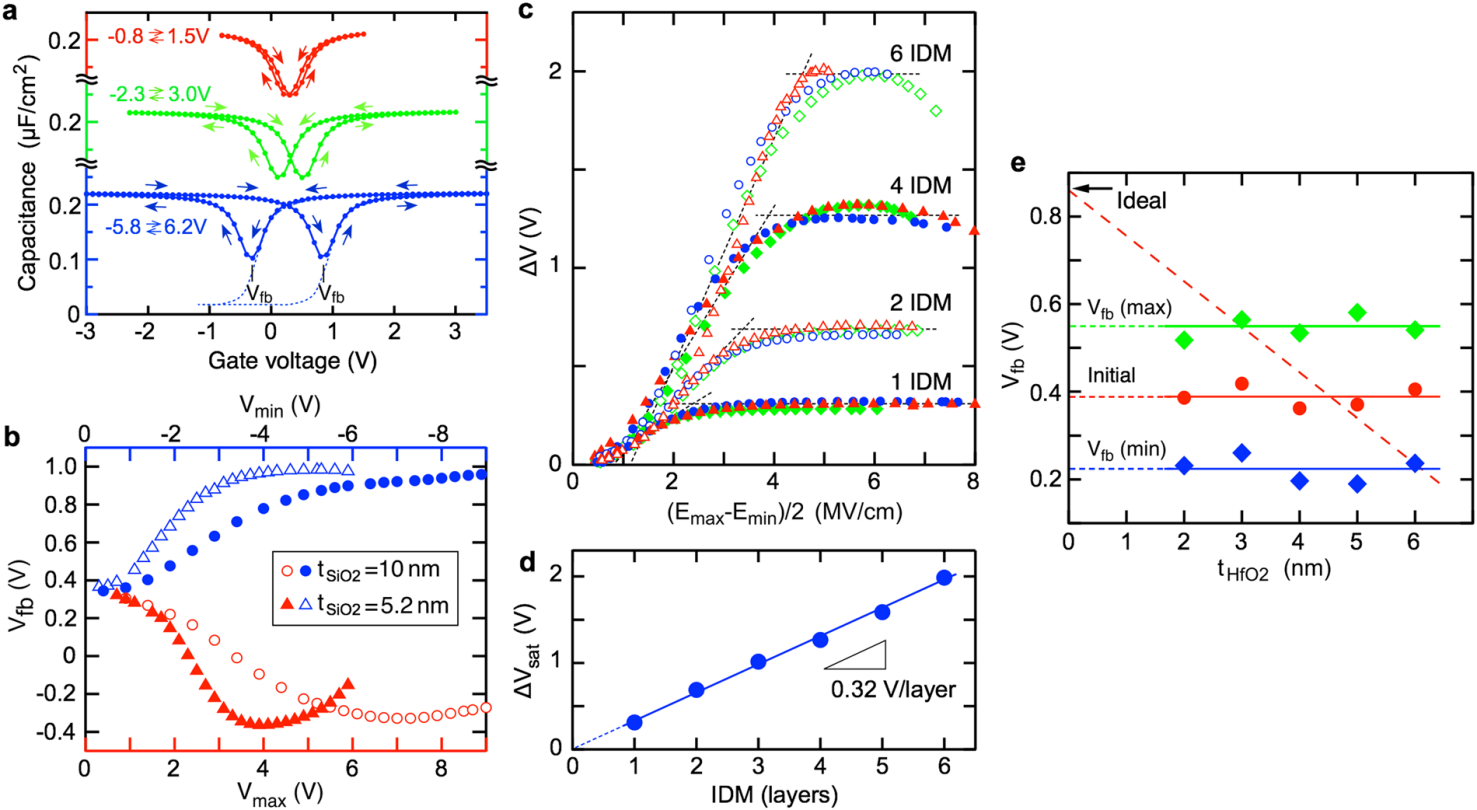
Hysteresis *C-V* characteristics of HfO_2_/SiO_2_ IDM MOS capacitors. **(a**) *C-V* curves of the four-stacked IDM MOS capacitor consisting of 3.5-nm-thick top HfO_2_, 1.8-nm-thick inner SiO_2_, 1.8-nm-thick inner HfO_2_, and 10-nm-thick bottom SiO_2_ layers. Double sweep measurements are performed at 5 kHz under weak light illumination. Sufficient electric fields for both positive and negative bias ranges are produced in the oxide layers. (**b**) Bias dependence of the flat-band voltage (*V*_*fb*_) observed from the four-stacked IDM MOS capacitor. Sample with a thin bottom SiO_2_ layer (5.2 nm) exhibits a *V*_*fb*_ shift in the lower voltage range compared to a thick bottom SiO_2_ layer (10 nm). This difference is almost canceled out in the electric field dependence (Supplementary Fig. 3a), suggesting that the IDM operation is a field driven process. (**c**) Dependence of the modulation width on electric field. Modulation width (*ΔV*) is the difference between the *V*_*fb*_ values of the forward and backward voltage sweeps. (**d**) Dependence of the saturation modulation width, *ΔV*_*sat*_, on the number of IDM layers. The TiO_2_ modulator has an ability to modulate the interface dipole by 0.32 V. (**e**) Dipole modulation width of a single IDM structure as a function of HfO_2_ thickness. Initial *V*_*fb*_, maximum *V*_*fb*_, and minimum *V*_*fb*_ values are not affected by the top HfO_2_ thickness (t_HfO2_). This result suggests that the change in the interface dipole dominates the observed voltage shifts.

The hysteresis *C-V* curves show that the voltage shift in the forward sweep is smaller than that in the backward sweep (Fig. 2c). This tendency is mainly due to the depletion of minority carriers in the negative voltage range. That is, the Si depletion layer prevents the generation of a sufficient electric field in the oxide layers. To investigate the IDM behavior in both polarity ranges, lower-frequency *C-V* curves were measured under a weak light illumination, which generates sufficient minority carriers. Approximately symmetric shifts of the flat-band voltage (*V*_*fb*_) are observed for both polarities (Fig. 3a and b).

In the case of backward sweeping (*i*.*e*., after positive bias stress), the turn-back behavior is recognized for the thinner bottom SiO_2_ sample, suggesting that electron injection from Si into the IDM structure through the bottom SiO_2_ layer occurs similar to a flash memory^39^. The *V*_*fb*_ shift of the thinner bottom SiO_2_ sample occurs in the lower voltage region compared to the thicker bottom SiO_2_ sample. However, the plot as a function of the electric field agrees well with the observations (Supplementary Fig. S3a). Various IDM structures with different bottom SiO_2_ layers (5–10 nm) show consistent electric-field dependences (Fig. 3c). This result suggests that the dipole modulation is an electric field driven phenomenon. The saturated hysteresis in the high electric field region (*i*.*e*., the maximum modulation width) is roughly proportional to the number of IDM layers, assuming the average estimated modulation capability of a single IDM layer is 0.32 V.

It is worth describing the behavior of a single IDM structure to understand the observed hysteresis characteristics. The initial *V*_*fb*_ values, which were measured before applying a high electric field, are almost independent of the top HfO_2_ thickness (Fig. 3e). Compared to the ideal *V*_*fb*_ value estimated from the work function difference between Si and the Ir gate metal [*Φ*_*MS*_ (V)], a negative voltage shift takes place. Here we ignore the fixed charges and dipoles in the bottom SiO_2_/Si structure according to the previous studies^28–30^. For simplicity, we assume two types of charges at the HfO_2_/1ML-TiO_2_/SiO_2_ interfaces: a positive sheet charge [*S*_*I*_ (cm^−2^)] and a dipole layer with a negative sheet charge on the HfO_2_ side and positive on the SiO_2_ side [*Φ*_*D*_ (V)]. The dependence of *V*_*fb*_ on HfO_2_ thickness ${t}_{{\mathrm{Hfo}}_{2}}$ (nm)] is expressed as^28,40^

1 $${V}_{FB}={\Phi }_{MS}-\frac{q{S}_{I}{t}_{HfO2}}{{\varepsilon }_{HfO2}}-{\Phi }_{D},$$

where ε_*HfO2*_ is the dielectric constant of the HfO_2_ layer. Equation (1) indicates that *V*_*fb*_ should be proportional to the HfO_2_ thickness when the unipolar charges dominate the voltage shifts, as shown by the dashed line in Fig. 3e. The observed *t*_*HfO2*_ dependence implies that the initial voltage shift is dominated by the interface dipole. The estimated initial dipole strength is about 0.47 V, which is slightly larger than that of the HfO_2_/SiO_2_ interface^26,28^. The maximum and minimum *V*_*fb*_ shifts after applying a high electric field are independent of the HfO_2_ thickness, suggesting that the observed field-induced voltage shift is due to the change in dipole strength. It is concluded that the interface dipole in the HfO_2_/1-ML TiO_2_/SiO_2_ IDM system of 0.47 V is modulated by about ± 0.16 V.

**Figure 4:**
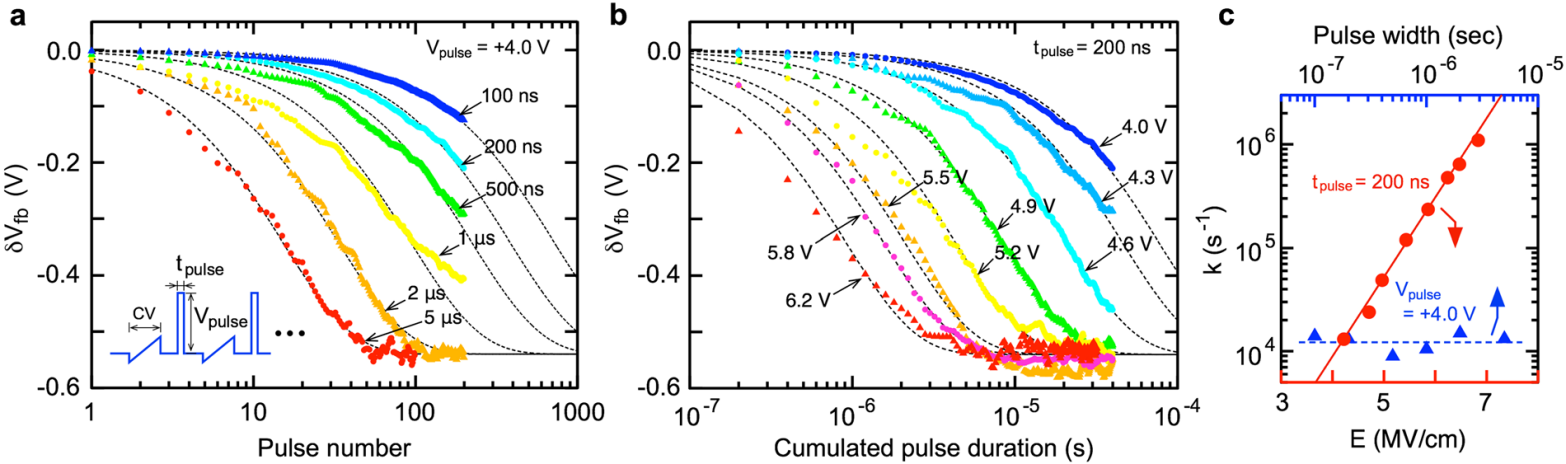
Pulse response characteristics of the four-stacked HfO_2_/SiO_2_ IDM MOS capacitor. IDM MOS capacitor consists of 3.5-nm-thick top HfO_2_, 1.8-nm-thick inner SiO_2_, 1.8-nm-thick inner HfO_2_, and 5-nm-thick bottom SiO_2_ layers. (**a**) *V*_*fb*_ shift with various pulse widths and pulse voltage of +4.0 V. The plotted *δV*_*fb*_ values show the voltage shifts from the initial *V*_*fb*_. (**b**) *V*_*fb*_ shifts with various pulse voltages and a pulse width of 200 nsec. Dotted lines in Fig. 4a,b show the fitting curves based on the random bond breakage/repair model. (**c**) Reaction rate (*k*) as functions of electric field and pulse width.

The pulse response is an important characteristic when discussing the modulation mechanism as well as when applying it to memory and synaptic devices. In this study, we examined the pulse-induced *V*_*fb*_ shift using a repetitive sequence of the *C-V* measurement and pulse application (Fig. 4a, inset). Since the six-stacked IDM structure shows a slightly larger charge trapping effect under a high electric field (Fig. 3c), the four-stacked IDM structure was investigated to examine the IDM pulse response. Figure 4a and b exhibit the strong dependence of the voltage shift on the pulse width [*t*_*pulse*_ (sec)] and the pulse voltage [*V*_*pulse*_ (V)].

A promising IDM mechanism is the electric-field-induced breakage/repair of the interfacial Ti-O bonds. More simplistically, the bistable switchable state between the broken Ti-O and the repaired Ti-O bonds can be assumed. In other words, the Ti coordination number changes due to the electric field (*e*.*g*., between the five-fold and four-fold Ti atom). The thermochemical theory proposed for the breakage of Si-O bonds^41,42^ is a sophisticated expression to explain the electric-field-induced bond breakage. The reaction rate, *k* (s^−1^), under the electric field, *E* (V/cm), is given by

2 $$k={v}_{0}exp(-\frac{{\rm{\Delta }}{H}_{0}^{\ast }-{p}_{eff}E}{{k}_{B}T}),$$

where *v*_0_ is the molecular vibrational frequency, which is generally on the order of ~10^13^ (s^−1^). *T* and *k*_*B*_ are the temperature (K) and Boltzmann’s constant, respectively. Zero-field activation energy [*ΔH*_0_ (eV)] exponentially decreases the reaction rate, while the effective dipole moment [*p*_*eff*_ (eÅ)] dominates the electric-field dependence. Hence, these two parameters can be separately deduced from the field dependence of the reaction rate, as described below.

At an actual IDM interface, the large amount of Ti-O bonds (~10^14^ cm^−2^) contributes to the modulation. In this discussion, we assume the simplest kinetics where the bond breakage/repair process proceeds randomly. This means that the nucleation and domain growth, which are general polarization switching kinetics in ferroelectric films, are neglected^43–45^. The total amount of bonds, *θ* (cm^−2^), which suffer from the breakage/repair process from 0 seconds to the specific time, *t* (sec), follows the rate equation: *dθ/d t* = (1* − θ*) · *k*. Thus, the amount of switched bonds can be given by *θ*(*t*) = 1 − *exp*(*−k* · *t*). After applying a suitable electric field for a sufficient time, *δV*_*fb*_ reaches the saturated voltage [*δV*_*sat*_ (V)]. The measured time dependence should be expressed as *δV*_*fb*_ (*t*) = *δV*_*sat*_ · [1* − exp*(*−k* · *t*)]. This equation has a good consistency with the measured data, and the reaction rate can be deduced as shown by Fig. 4c.

Significant change is not recognized from the pulse-width dependence shown in Fig. 4c, which suggests that the effect of different time-dependent phenomena such as trap and dipole loss responses^34,35^ are not significant in this pulse-width range. From the field dependence shown in Fig. 4c, *ΔH*_0_ and *p*_*eff*_ are estimated to be 0.72 eV and 4.6 eÅ, respectively. These parameters are within an acceptable range. For example, the reported bond breakage of O−Si≡O_3_ tetragonal molecules in silica is *ΔH*_0_ = 1–2 eV and *p*_*eff*_ = 7–13 eÅ, depending on the charge trapping, bond distortion, and the defective structure^41,42^. It has also been reported that Hf-O bond breakage in HfO_2_ shows *ΔH*_0_ = 4.6 eV^46^. Obviously, *ΔH*_0_ of the IDM operation is smaller than those of Si-O and Hf-O bond breakages. In addition, the reported breakdown field (*Ε*_*bd*_) of TiO_2_ is 1–2.5 MV/cm, which is smaller than either SiO_2_ (15 MV/cm) and HfO_2_ (6.7 MV/cm)^42,46,47^. Therefore, we can reasonably consider that the Ti-O bond in the IDM layer is easily broken compared to the Si-O and Hf-O bonds. The displacement of the charged Ti and O atoms associated with the Ti-O bond breakage likely alters the interface dipole of the HfO_2_/1-ML TiO_2_/SiO_2_ structure, as described for HfO_2_/1-ML TiO_2_/Si structure in Fig. 1a. In the amorphous HfO_2_/1-ML TiO_2_/SiO_2_ structure, various Ti-O bonding configurations may contribute to the IDM operation because such an operation occurs at the amorphous atomically rough interface. However, more elaborate studies are necessary to assign detailed structural changes.

**Figure 5:**
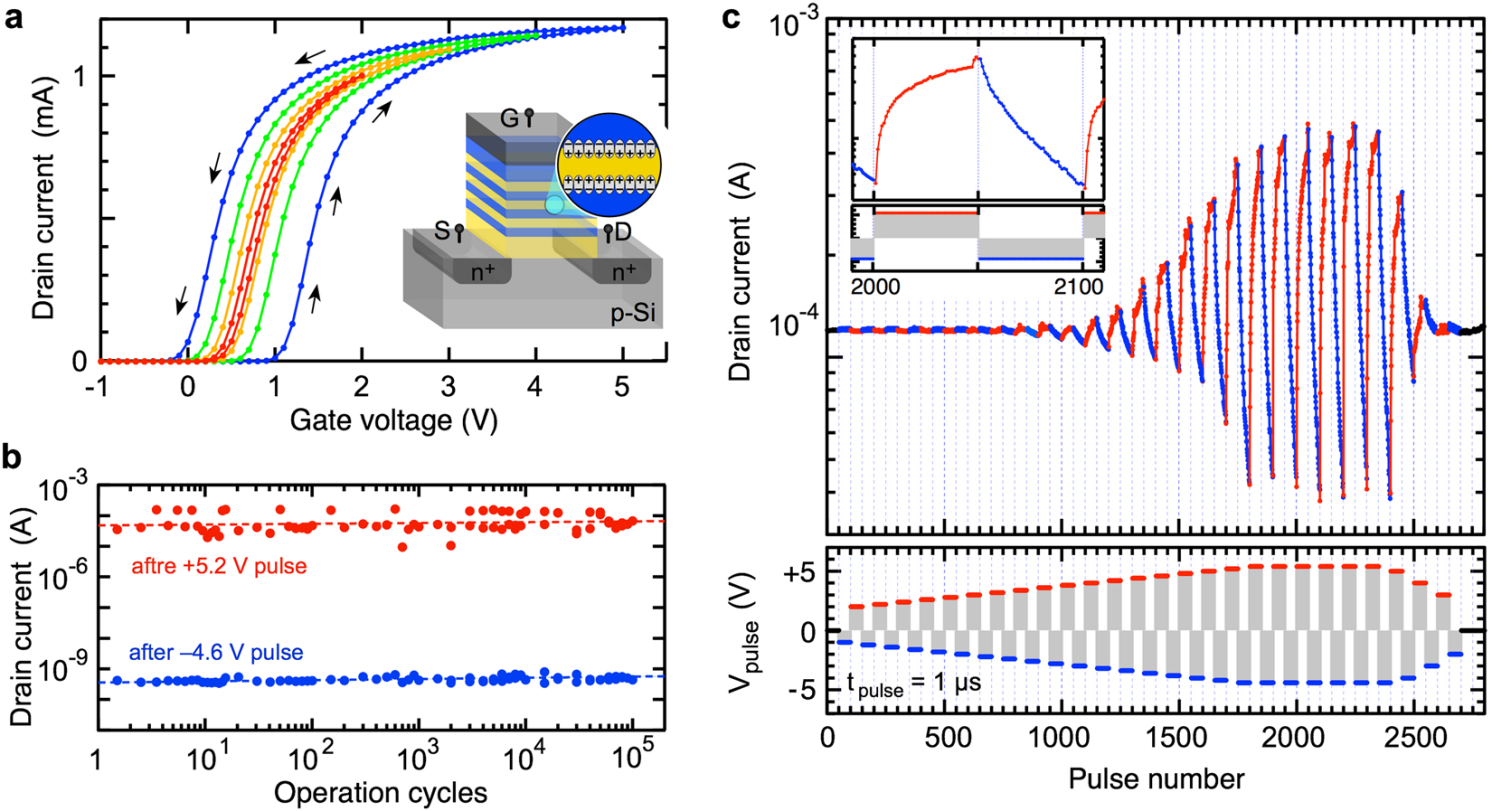
Operation of six-stacked HfO_2_/SiO_2_ IDM FET. IDM FET consists of 3.5-nm-thick top HfO_2_, 1.8-nm-thick inner SiO_2_, 1.8-nm-thick inner HfO_2_, and 5-nm-thick bottom SiO_2_ layers. (**a**) Drain current *vs*. gate voltage (*I*_*d*_ − *V*_*g*_).  The drain current of the IDM FET were measured at *V*_*ds*_ = 0.3 V in various gate voltage ranges: −1 V $\rightleftarrows$ +2 V, −2 V$\rightleftarrows$ +3 V, −3 V $\rightleftarrows$ +4 V, and −4 V $\rightleftarrows$ +5 V. (**b**) Cyclic switching characteristics. Small and large *I*_*d*_ states are switched by alternatively applying 100-μsec pulses at +5.2 V and −4.6 V. (**c**) Pulse-induced continuous current change. Drain current is monitored at *V*_*ds*_ = +0.3 V and *V*_*g*_ = +0.3 V by applying 1-μsec pulses with various gate voltages. Interface dipoles hardly respond to low absolute voltage pulses (−2.5 V < *V*_*pulse*_ < +3.5 V) and become sensitive to the increase in the absolute voltage outside this range.

An appropriate hysteresis is observed in the *I*_*d*_ − *V*_*g*_ curve of the FET device with six-stacked HfO_2_/SiO_2_ IDM structure (Fig. 5a), demonstrating that the IDM phenomena can be read as the channel current. The endurance characteristics observed by alternately applying positive and negative bias pulses show good cyclic switching of 10^5^ and a large current difference of about 10^5^. However, this memory performance has yet to reach a level to be competitive with advanced HfO_2_ FeFET and two-terminal resistance change devices^3–8,48,49^. In particular, the large operation voltage is a serious issue.

We consider that scaling of the equivalent oxide thickness (EOT) is indispensable to suppress the operating voltage because the current multi-stack IDM gate is not optimized in terms of the EOT scaling. Below are prospective ideas for the EOT scaling. (1) Bottom dielectric layer: the 5–10-nm thick SiO_2_ bottom layer is employed in the current IDM structures. A thin HfO_2_ layer should be effective, provided that a low *D*_*it*_ can be maintained. (2) Scaling of inner dielectric layers: the current multi-stack IDM structure employs 1.8-nm thick HfO_2_ and SiO_2_ layers. Thinning these layers is possible and effective, but the interface abruptness should be simultaneously improved. (3) Material selection of IDM dielectrics: the IDM structure using dipoles induced at high-*k*/high-*k* interfaces is advantageous compared to the current low-dielectric-constant SiO_2_/HfO_2_ interface. We expect to realize low-voltage IDM operations using (1)–(3).

Furthermore, we would like to mention an issue with the multi-stack IDM structure, which is shown in Fig. S3c. The retention characteristics are degraded more than the HfO_2_/Si IDM structure (Fig. 1d). The depolarization field and the carrier traps are considered to be the major causes^50,51^. For the latter, further studies on the oxide material selection and formation method to eliminate the defects in multi-stack oxide structure are necessary.

The pulse-induced current change was investigated as an application to synaptic devices (Fig. 5c). The current is approximately constant at pulse voltages between −2.5 V and +3.5 V. Outside this range the current change becomes large as the absolute pulse voltage increases. This behavior can be easily understood by the above thermochemical mechanism represented by Eq. (2). An important feature of this mechanism is the threshold voltage, which is an important function in the STDP operation^16,52^. Furthermore, these current changes can be roughly predicted by the above random bond breakage/repair model, which is advantageous in designing synaptic device. STDP operations based on a similar three-terminal FeFET devices have been reported, where the current can be controlled gradually by rectangular or triangular pulses^17,53^. We expect that the STDP operation of IDM FET device can be realized in a similar manner.

In conclusion, the concept of an IDM memory is proposed and demonstrated using HfO_2_-based gate stacks of Si MOS devices. In this demonstration, the 1-ML TiO_2_ modulator inserted at HfO_2_/Si and HfO_2_/SiO_2_ interfaces plays a major role. The electrical characteristics of multi-stacked HfO_2_/1-ML TiO_2_/SiO_2_ IDM MOS capacitors are investigated in detail because this IDM structure is preferable for Si-FET-based flash memory devices. After fabricating the multi-stacked IDM FET device, its switching operation and pulse-induced current change are presented.

## Methods

### Oxide deposition

HfO_2_, SiO_2_, and TiO_2_ films were deposited via an ultra-high vacuum evaporation system^30^. Metallic Hf, Si, and Ti were evaporated in oxygen pressure without heating Si substrate. After the formation of HfO_2_/TiO_2_/Si and HfO_2_/TiO_2_/SiO_2_/Si IDM structures, post-deposition annealing was performed at 350–400 °C for 1 min. The x-ray photoelectron spectroscopy confirmed that almost stoichiometric amounts of HfO_2_, SiO_2_, and TiO_2_ were deposited on the substrates by this method. In particular, this evaporation method allows the HfO_2_/Si interface with a monolayer thickness to be controlled^28–30,32^ and is indispensable to prepare the HfO_2_/1-ML TiO_2_/Si structure, which was employed in the first demonstration of the IDM concept.

### MOS and FET fabrication

The MOS capacitors were prepared on n-type or p-type Si(100) substrates. The bottom SiO_2_ layers were grown by thermal oxidation of Si substrates. SiO_2_ layers with thicknesses between 5–10 nm were fabricated by hydrofluoric acid etching. For the single IDM structures, top HfO_2_ layers of varying thicknesses between 2–6 nm were deposited. For the multi-stack IDM structures, 1.8-nm-thick inner HfO_2_, 1.8-nm-thick inner SiO_2_, and 3.5-nm-thick top HfO_2_ layers were deposited. Ir films were deposited on the oxide layers, and the Ir electrodes (100 × 100 μm) were fabricated by a lithography technique. The MOS FET devices (L_g_ = 1 μm and W = 100 μm) were fabricated by the so-called gate last processes^32^. First, the source/drain regions were fabricated on p-type Si(100) substrates. Second, multi-stack IDM structures with the same thick layers as the above MOS capacitors were fabricated. Finally, the Ir gate electrodes were fabricated by the same method used to prepare the MOS capacitors.

### *C-V* measurements and analyses

The high-frequency *C-V* curves were measured at 1 MHz with a gate voltage sweep from a negative to a positive bias, and then immediately swept in the opposite direction. The lower-frequency *C-V* curves were measured at 5 kHz under weak light irradiation. The flat-band voltage (*V*_*fb*_) was determined by fitting with the ideal *C-V* curve (Fig. 3a). It is difficult to adopt the same method to the *C-V* curves of HfO_2_/Si IDM MOS capacitors due to the large stretched characteristics. Figure 1c plots the relative voltage shifts determined by the flat-band capacitor^34,35^. The maximum and minimum electric fields were estimated from the measured maximum capacitances in the accumulation and inversion ranges, respectively.

### Pulse response measurements

First, a gate voltage of −4 V was applied as an initialization, shifting *V*_*fb*_ to the positive bias direction. Second, 100-times cyclic *C-V* measurements were performed at 1 MHz in the range from −0.4 V to +0.7 V. After this process, *V*_*fb*_ was stabilized between 0.35–0.45 V, which is the stable state under these *C-V* measurement conditions, and is defined as the initial *V*_*fb*_ (t = 0). Finally, the cyclic sequence of the *C-V* measurement under the same conditions and pulse application was performed (Fig. 4a, inset). The pulse electric field was determined for the initial state by using an ideal MOS *C-V* curve.

## Electronic supplementary material

Supplementary information
