# Supplementary material for: Electric-field-controlled interface dipole modulation for Si-based memory devices
Source: Sci Rep. 2018 May 31;8:8486. doi: 10.1038/s41598-018-26692-y (PMC5981312; doi:10.1038/s41598-018-26692-y)
Supplement: Supplementary file 1 — Supplementary information [file 41598_2018_26692_MOESM1_ESM.docx]

**[Supplementary information]**

**Electric-field-controlled interface dipole modulation for Si-based memory devices**

Noriyuki Miyata

National Institute of Advanced Industrial Science and Technology (AIST), Central 5, 1-1-1 Higashi, Tsukuba, Ibaraki 305-8565, JAPAN

1. **Physical analysis of the TiO_2_ modulator**

The HfO_2_/1-ML TiO_2_/Si structure in a MOS capacitor exhibiting an IDM behavior was observed by transmission electron microscopy (TEM). The TEM images exhibit a several-monolayer-thick bright region between the HfO_2_ film and the Si substrate (Fig. S1a). The HfO_2_/Si structure prepared by the same method without inserting a TiO_2_ modulator does not show such an interface region [1]. Thus, we conclude that the bright region corresponds to the TiO_2_ modulator, which probably related to Si-O-Ti-O bonding.

X-ray photoelectron spectroscopy (XPS) was used to identify the Ti oxidation states. A thin HfO_2_ layer (~1.5 nm) was deposited on a 1-ML TiO_2_/Si structure because a thick surface HfO_2_ layer prevents detection of the photoelectron electrons emitted from the buried TiO_2_ layer. The Ti *2p* photoelectron spectrum (I) in Fig. 1b exhibits Ti^4+^ and Ti^3+^ oxidation components, which are separated by assuming the previously reported parameters [2,3]. The Ti^4+^ oxidation state (TiO_2_) is the major component. With regard to the Ti^3+^ component, the ratio should be carefully considered as the fitting curve largely depends on the fitting parameters (*i.e.*, the chemical shift and peak width energies). The 1-ML TiO_2_ modulator at the SiO_2_/HfO_2_ interface was also examined by XPS. A 1.8-nm-SiO_2_/1-ML TiO_2_/2-nm-HfO_2_ stack structure was prepared on a thick Ir film surface. The Ti 2*p* photoelectron spectrum observed in Fig. 1b (II) suggests the TiO_2_ modulators at both HfO_2_/Si and HfO_2_/SiO_2_ interfaces have similar chemical structures. The Hf 4*f* photoelectron spectra were also measured. They indicate that our method forms an approximately stoichiometric HfO_2_ structure. This is consistent with our previous results [4]. The Si 2*p* photoelectron spectrum observed from the 1.8-nm-SiO_2_/1-ML TiO_2_/2-nm-HfO_2_ stack structure demonstrates that the Si^4+^ oxidation state (SiO_2_) is the major chemical structure. Of course, the sub-oxide components in the HfO_2_, TiO_2_, and SiO_2_ layers (*i.e.*, defective structures) cannot be excluded from the XPS study. Presently, the effect of such minor components on the IDM operation is unclear.


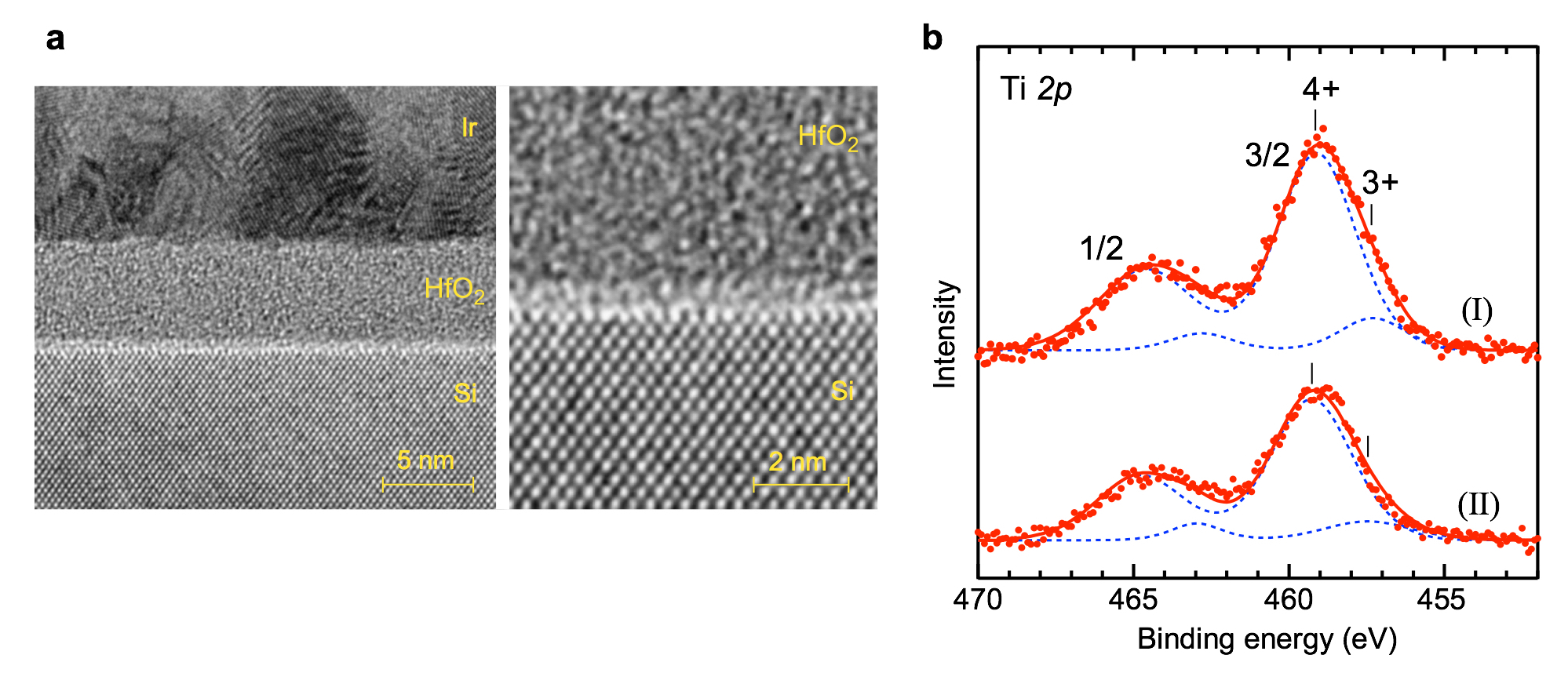


**Figure S1 | Structural characteristics of the 1-ML TiO_2_ modulator.** **a**, Transmission electron microscopy (TEM) images of the 5.5-nm-thick HfO_2_/1-ML TiO_2_/Si IDM MOS structure. Monolayer thick bright region at the HfO_2_/Si interface corresponds to the TiO_2_ layer. **b**, Ti 2*p* photoelectron spectra of 1-ML TiO_2_ modulators. (I) 1.5-nm-thick HfO_2_/1-ML TiO_2_/Si structure and (II) 1.8-nm-thick SiO_2_/1-ML TiO_2_/2-nm-HfO_2_ structure on thick Ir films.

1. **Conductance measurement and interface charge traps**

To investigate the electrical charge traps around oxide/Si interfaces, conductance measurements of the MOS capacitors prepared on both n-type and p-type Si(100) substrates were performed. The conductance maps (*G_p_/ω*) of the 5.5-nm thick HfO_2_/1-ML TiO_2_/Si structures (Fig. S2a) show that large gate voltages (>3 V) are required to shift the conductance peak from 100 Hz to 1 MHz, indicating that modulation of the Si surface potential is hindered by the interface charge traps [5]. This result suggests that the HfO_2_/1-ML TiO_2_/Si structure is unsuitable for FET operations. On the other hand, the 3-nm-HfO_2_/1-ML TiO_2_/5-nm-SiO_2_/Si MOS capacitors show effective surface potential modulations (Fig. S2b). A small gate voltage (~0.3 V) is sufficient to shift the conductance peak in the same frequency range. This MOS interface quality is similar to the thermally grown SiO_2_/Si structures employed in standard CMOS devices.

Actually, the IDM FET shows good *I_d_-V_g_* characteristics (Fig. 5). The *D_it_* values were estimated from the measured conductance peaks, *(G_p_/ω)_peak_*, using the standard equation: *D_it_*=2.5・[(*G_p_/ω*)_peak_/*q*] [4]. The *D_it_* distribution shown in Fig. S2c indicates that a large amount of charge traps (>1x10^13^ cm^-2^ eV^-1^ around the mid-gap energy) exists at the HfO_2_/TiO_2_/Si MOS interface, but the charge traps are effectively suppressed in the HfO_2_/TiO_2_/SiO_2_/Si MOS structure. We should note that HfO_2_/Si MOS interface without inserting a TiO_2_ modulator maintains a low trap density (*D_it_*~2x10^11^ cm^-2^ eV^-1^ around the mid-gap energy) [6]. Therefore, the charge traps in the HfO_2_/1-ML TiO_2_/Si structure are attributed to the interface TiO_2_ modulator.


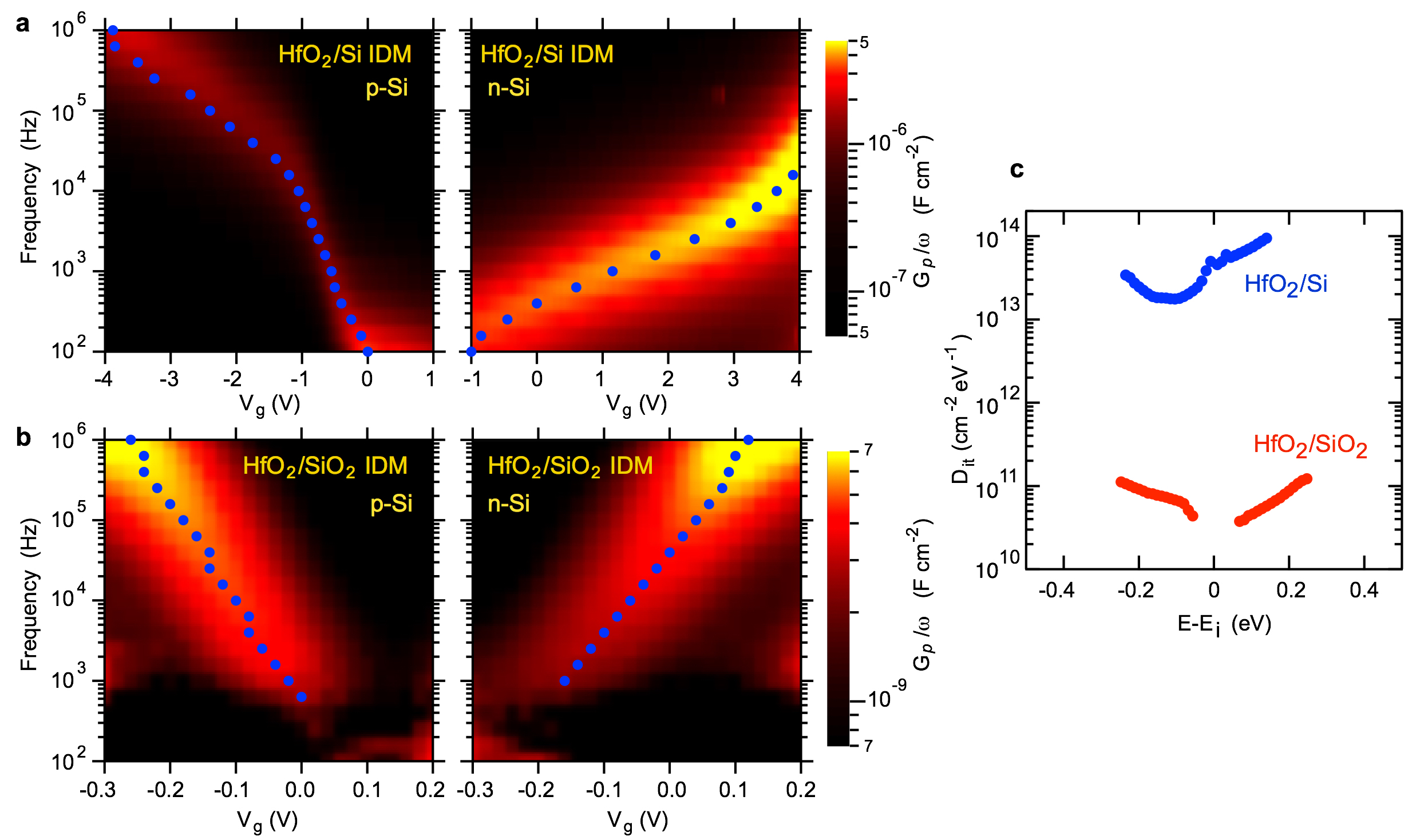


**Figure S2 | Conductance measurements of HfO_2_/Si and HfO_2_/SiO_2_ IDM MOS capacitors. a**, Conductance maps of 5.5-nm-HfO_2_/1-ML TiO_2_/p-Si and /n-Si MOS capacitors. **b**, Conductance maps of 3-nm-HfO_2_/1-ML TiO_2_/5-nm-SiO_2_/p-Si and n-Si MOS capacitors. **c**, Interface state densities (*D_it_*) estimated from the conductance data shown in Figs. S2a and S2b.

1. **Electrical characteristics of HfO_2_/SiO_2_-based IDM MOS capacitors**

The *V_g_* dependence of the *V_fb_* shift (Fig. 3b) depends on the thickness of the bottom SiO_2_ layer, although the same four-stacked IDM structures are fabricated. The *E_ox_* dependence of the *V_fb_* shift in Fig. S3a shows a good consistency between these two samples, suggesting that the dipole modulation is an electric-field driven phenomenon. In the graph, *E_min_* and *E_max_* correspond to the electric fields produced when a minimum negative voltage and a maximum positive voltage are applied, respectively. The electric field, (*E_max_*-*E_min_*)/2, in Fig. 3c was calculated from these values. The modulation width (*ΔV*) in Fig. 3c was also estimated from the same measurement. *ΔV* corresponds to the *V_fb_* difference between the forward and backward voltage sweeps. The turn-back behavior observed from the red plots in Fig. S3a suggests that the electrons injected from the Si substrate to the IDM structure are trapped. The six-stacked IDM structure (Fig. 3c) had a larger trapping effect compared to the four-stacked IDM structure. On the other hand, the single IDM structure (Fig. S3b) has a smaller effect. These results suggest that the TiO_2_ modulators at the HfO_2_/SiO_2_ interfaces include large charge traps similar to that observed in the HfO_2_/Si IDM structure (Fig. S2). Figure S3b also indicates that the HfO_2_/SiO_2_ interface without inserting a TiO_2_ modulator does not display *V_fb_* hysteresis and the trapping effect can be neglected unless the electric field exceeds 8MV/cm. Figure S3c, which shows the time-dependent capacitance data of the six-stacked HfO_2_/SiO_2_ IDM MOS capacitor after applying +6V and –6V stress, reveals that the retention characteristics are worse than those of the HfO_2_/Si IDM structure. Based on the previously reported mechanisms for the retention degradation of ferroelectric memory devices [7, 8], we considered that the major origins of the degraded characteristics are the effects of the depolarization field and charge traps. With regard to charge traps, we cannot deny the formation of a large amount of defects around the amorphous HfO_2_/SiO_2_ interface. To realize a practical memory device, further studies on material selection and IDM interface engineering are required.


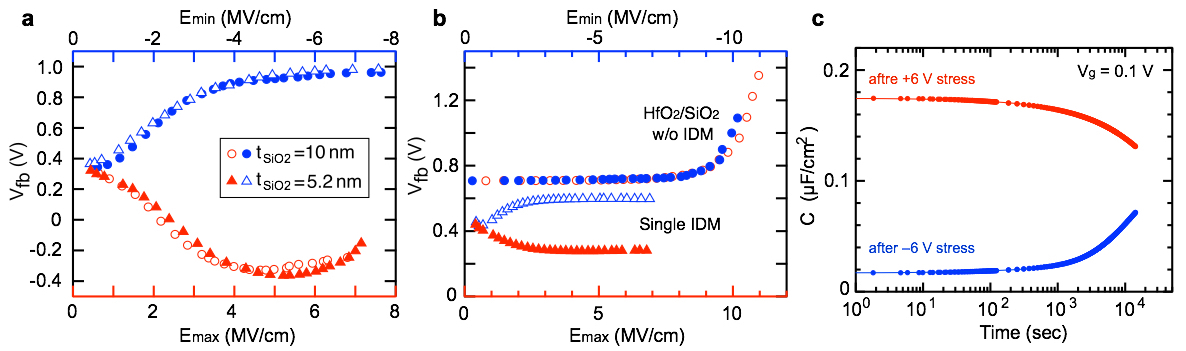


**Figure S3 | Electrical characteristics of HfO_2_/SiO_2_-based IDM MOS capacitors. a**, Electric-field dependence of the four-stacked HfO_2_/SiO_2_ IDM structure with different bottom interface SiO_2_ layers. Plotted *V_fb_* data are the same as those in Fig. 3b showing the gate-voltage dependence. The results support that dipole modulation is an electric-field-driven phenomenon. **b**, Electric-field dependence of single HfO_2_/SiO_2_ IDM and non-IDM HfO_2_/SiO_2_ structures. Data for the single 5-nm-HfO_2_/1-ML TiO_2_/10-nm-SiO_2_ IDM MOS capacitor are plotted. Conventional 5-nm-HfO_2_/10-nm-SiO_2_/Si stack structure without a TiO_2_ modulator is plotted as a reference, where IDM does not occur even after applying a high electric field (>5 MV/cm). The positive voltage shifts in the high electric field (> 8MV/cm) correspond to the effects of charge trapping. c, Retention characteristics of the six-stacked HfO_2_/SiO_2_ IDM structure with 10-nm-thick bottom SiO_2_ layer. Time dependence of MOS capacitance at 0 V after applying +6 and –6 V stress is shown.

**References**

[1] Miyata, N. Two-step behavior of initial oxidation at HfO_2_/Si interface. *Appl. Phys. Lett.* **89**, 102903 (2006).

[2] Bertaud, T. Sowinska, M. Walczyk, D. Thiess, S. Gloskovskii, A. Walczyk, C. & Schroeder, T. In-operando and non-destructive analysis of the resistive switching in the Ti/HfO_2_/TiN-based system by hard x-ray photoelectron spectroscopy. *Appl. Phys. Lett.* **101**, 143501 (2012).

[3] Calka, P. *et al*., Engineering of the Chemical Reactivity of the Ti/HfO_2_ Interface for RRAM: Experiment and Theory. *ACS Appl. Mater. Interfaces* **6**, 5056−5060 (2014).

[4] Miyata, N. Ichikawa, M. Nabarame, T. Horikawa, T. & Toriumi, A. Thermal stability of a thin HfO_2_/Ultrathin SiO_2_/Si structure: interfacial Si oxidation and silicidation. *Jpn. J. Appl. Phys.* **42**, L 138–L 140 (2003).

[5] Nicollian, E. H. & Brews, J. R. MOS (metal oxide semiconductor) Physics and Technology Wiley-Interscience Publication (1982)

[6] Miyata, N. Abe, Y. & Yasuda, T. Conductance Spectroscopy Study on Interface Electronic States of HfO_2_/Si Structures: Comparison with Interface Dipole. *Appl. Phys. Express* **2**, 035502 (2009).

[7] Ma, T. P. & Han, J.-P. Why is Nonvolatile Ferroelectric Memory Field-Effect Transistor Still Elusive?. *IEEE Electron Device Lett.* **23**, 386–388 (2002).

[8] Hoffman, J. *et al.* Ferroelectric field effect transistors for memory applications. *Adv. Mater.* **22**, 2957–2961 (2010).
